# Comparative transcript profiling of resistant and susceptible peanut post-harvest seeds in response to aflatoxin production by *Aspergillus flavus*

**DOI:** 10.1186/s12870-016-0738-z

**Published:** 2016-02-27

**Authors:** Houmiao Wang, Yong Lei, Liyun Wan, Liying Yan, Jianwei Lv, Xiaofeng Dai, Xiaoping Ren, Wei Guo, Huifang Jiang, Boshou Liao

**Affiliations:** Key Laboratory of Oil Crop Biology of the Ministry of Agriculture, Oil Crops Research Institute of Chinese Academy of Agricultural Sciences, Wuhan, 430062 China; Chinese Academy of Agricultural Sciences-International Crop Research Institute for the Semi-Arid Tropics Joint Laboratory for Groundnut Aflatoxin Management, Oil Crops Research Institute of Chinese Academy of Agricultural Sciences, Wuhan, 430062 China; Institute of Agro-Products Processing Science and Technology, Chinese Academy of Agricultural Sciences, Beijing, 100193 China

**Keywords:** *Arachis hypogaea*, Post-harvest resistance, Aflatoxin production, Transcriptome

## Abstract

**Background:**

Aflatoxin contamination caused by *Aspergillus flavus* in peanut (*Arachis hypogaea*) including in pre- and post-harvest stages seriously affects industry development and human health. Even though resistance to aflatoxin production in post-harvest peanut has been identified, its molecular mechanism has been poorly understood. To understand the mechanism of peanut response to aflatoxin production by *A. flavus*, RNA-seq was used for global transcriptome profiling of post-harvest seed of resistant (Zhonghua 6) and susceptible (Zhonghua 12) peanut genotypes under the fungus infection and aflatoxin production stress.

**Result:**

A total of 128.72 Gb of high-quality bases were generated and assembled into 128, 725 unigenes (average length 765 bp). About 62, 352 unigenes (48.43 %) were annotated in the NCBI non-redundant protein sequences, NCBI non-redundant nucleotide sequences, Swiss-Prot, KEGG Ortholog, Protein family, Gene Ontology, or eukaryotic Ortholog Groups database and more than 93 % of the unigenes were expressed in the samples. Among obtained 30, 143 differentially expressed unigenes (DEGs), 842 potential defense-related genes, including nucleotide binding site-leucine-rich repeat proteins, polygalacturonase inhibitor proteins, leucine-rich repeat receptor-like kinases, mitogen-activated protein kinase, transcription factors, ADP-ribosylation factors, pathogenesis-related proteins and crucial factors of other defense-related pathways, might contribute to peanut response to aflatoxin production. Notably, DEGs involved in phenylpropanoid-derived compounds biosynthetic pathway were induced to higher levels in the resistant genotype than in the susceptible one. Flavonoid, stilbenoid and phenylpropanoid biosynthesis pathways were enriched only in the resistant genotype.

**Conclusions:**

This study provided the first comprehensive analysis of transcriptome of post-harvest peanut seeds in response to aflatoxin production, and would contribute to better understanding of molecular interaction between peanut and *A. flavus*. The data generated in this study would be a valuable resource for genetic and genomic studies on crops resistance to aflatoxin contamination.

**Electronic supplementary material:**

The online version of this article (doi:10.1186/s12870-016-0738-z) contains supplementary material, which is available to authorized users.

## Background

Peanut (*Arachis hypogaea* L.) is an important cash and oilseed crop and a key source of vegetable oil and protein worldwide. However, aflatoxin contamination caused by *Aspergillus flavus* and/or *A. parasiticus* has been a serious constraint to peanut industry, which is of great concern because aflatoxins are toxic, carcinogenic and teratogenic compounds associated with both acute and chronic toxicity in animal and human [1, 2]. Infection of peanut by *A. flavus* occurs in both pre-harvest [3, 4] and post-harvest stages [5, 6]. With appropriate drying, storing, processing, transporting and monitoring, healthy peanuts harvested from normal growth conditions are processed into secure and nutritious products for human/animal consumption. Unfortunately, farmers in many developing countries in Asia and Africa, can’t afford the cost associated with prevention, monitoring and mitigation of aflatoxin in peanut food/feed. Post-harvest aflatoxin contamination has led to an increased risk of exposure to aflatoxin resulting in outbreaks of acute aflatoxin poisoning [7] and increased morbidity in children suffering from stunted growth and malnutrition [8–10]. In addition, post-harvest aflatoxin contamination incurs significant economic costs, such as produce and market value losses, health care and associated disease surveillance, and for monitoring and mitigation of aflatoxin in peanut commodities [2, 11]. Thus, post-harvest aflatoxin contamination is an intractable problem in peanut products. Several management practices, including proper storage and transportation conditions, strict monitoring measures, and breeding cultivars for resistance to biotic and abiotic stresses, could prevent and/or reduce post-harvest aflatoxin contamination. Improvement of resistance to *A. flavus* invasion and/or aflatoxin production in peanut is considered to be the most cost-effective management approach. However, the resistance to post-harvest aflatoxin contamination in peanut hasn’t been well understood.

The mycelia of *A. flavus* have to penetrate the peanut shell and seed coat before they reach the nutrient-rich cotyledons to derive sustenance. Resistance to aflatoxin contamination in peanut could be broadly classified into pod infection (shell), seed invasion (seed coat) and aflatoxin production (cotyledon) [12]. The first interaction between *A. flavus* and peanut is at the pod shell, which is a physical barrier, and the resistance is attributed to the shell structure. For post-harvest peanut, the resistance to pod infection is limited practical value, because ease of shelling is an important consideration in peanut industry. Moreover, the resistance of the pod shell to *A. flavus* infection would disappear when the shell is damaged or the peanut is shelled. The second barrier to this fungus is the seed coat, whose thickness, density of palisade layers, wax layers, and absence of fissures and cavities, are major contributors to the resistance to seed invasion. However, the seed coat would fail to resist *A. flavus* invasion when the testa is damaged or decorticated. *A. flavus* ultimately colonizes the cotyledons in the seed and produces the aflatoxin. Resistance to aflatoxin production is a very complex defensive mechanism affected by various biotic and abiotic factors. However, this kind of resistance to aflatoxin production, including the stress-responsive mechanism, is persistent and active [13, 14]. To develop effective measures to combat post-harvest aflatoxin contamination, it is important to investigate the molecular mechanisms of peanut resistance to aflatoxin production.

RNA-sequencing (RNA-seq) is a powerful and cost-efficient high-throughput technology for transcriptomic profiling that has been used successfully to interrogate the transcriptome of peanut in different development stages and response to various stresses [15–20]. With its higher sensitivity, RNA-seq could efficiently detect a larger range of dynamically expressed genes than microarrays. Furthermore, RNA-seq has been used to survey sequence variations and complex transcriptomes with low false-positive rates, and reproducibility [21]. Application of this technology has greatly accelerated understanding of the complexity of gene expression, regulation and networks [21], and has shown immense potential in explaining the molecular mechanism of host-resistance against pathogen infection. Peanut’s resistance to *Aspergillus* colonization/aflatoxin production has been extensively reported, indicating that peanut has evolved a series of defense mechanisms against the fungi [22]. However, molecular mechanism of peanut resistance to aflatoxin production by *A. flavus* has been obscure.

To gain a comprehensive understanding of the molecular mechanism of resistance to aflatoxin production in post-harvest peanut seed, we used RNA-seq to obtain and compare transcriptomic profiles of a resistant genotype Zhonghua 6 and a susceptible genotype Zhonghua 12 in post-harvest seeds, with and without *A. flavus* inoculation, at the whole-genome level. *De novo* transcriptome assembly, functional annotation, and analysis of specific transcripts related to peanut’s response to aflatoxin production by *A. flavus* were implemented. Differentially expressed genes and metabolic pathways associated with resistance to aflatoxin production were revealed by comparing *A. flavus*-inoculated and non-inoculated seeds of the resistant/susceptible peanut genotypes. A better understanding of the molecular mechanism of resistance to aflatoxin production would aid in improving strategies to develop new resistant peanut cultivars. In addition, the transcriptomic information would aid functional genomics studies and further the understanding of resistant mechanisms to aflatoxin contamination in crops.

## Results

### Comparison of aflatoxin production in post-harvest peanut seeds with fungal colonization

The aflatoxin content was quantified to define the response of Zhonghua 6 (resistant, R) and Zhonghua 12 (susceptible, S) to aflatoxin production by *A. flavus*. Aflatoxin was not tested neither in R nor S on the 1^st^ day after incubation, and was tested starting from the 2^nd^ day after incubation in both R and S genotypes. The aflatoxin content increased significantly both in R and S after the 2^nd^ day after incubation; however, the trend of aflatoxin accumulation varied in the R and S genotypes (Table 1). In the R, the aflatoxin content increased most quickly between the 3^rd^and 4^th^ day after incubation and then the increase ratio slowed down and the content became stable after the 7^th^ day. In the S, the aflatoxin content increased rapidly from the 3^rd^ to the 7^th^ day after incubation and then also remained stable. The aflatoxin content in the R was far lower than that in the S from the 2^nd^ day. At the peak of aflatoxin accumulation, the content in the S was over 10-folds of that in the R. Meanwhile, aflatoxin was not detected in non-inoculated R and S samples at all the 10 time points (Table 1). From the above experiment, the R possessed a desirable resistance to aflatoxin production in post-harvest seeds, while the S was highly susceptible.

**Table 1:** The dynamic changes of aflatoxin content in the resistant genotype Zhonghua 6 and susceptible Zhonghua 12 during *A. flavus* colonization

Cultural time (d)	Aflatoxin content in Zhonghua 6 (μg/kg)		Aflatoxin content in Zhonghua12 (μg/kg)	
	CK	T	CK	T
1	0	0 ± 0	0	0 ± 0
2	0	1130.2 ± 104.6	0	4462.8 ± 236.9
3	0	3175.5 ± 232.8	0	12687.1 ± 720.2
4	0	12609.8 ± 1226.4	0	76671.9 ± 6401.5
5	0	16906.0 ± 1311.6	0	111040.6 ± 10125.6
6	0	19156.9 ± 1608.0	0	140227.3 ± 11256.9
7	0	21107.6 ± 1487.4	0	195223.8 ± 14354.4
8	0	21012.0 ± 1441.2	0	202425.0 ± 14709.6
9	0	21059.8 ± 1197.6	0	193510.8 ± 14805.0
10	0	20180.4 ± 1501.8	0	202632.5 ± 14385.6

*T* the peanut seed with inoculated *A. flavus*, *CK* the peanut seed without inoculated *A. flavus*

### Transcriptome sequencing and *de novo* assembly

The above aflatoxin content results suggested that peanut might alter their gene expression in response to aflatoxin production by *A. flavus* during incubation. The 1^st^, 3^rd^ and 7^th^ day after incubation were chosen as the inflection time points to study the defensive molecular metabolism of post-harvest seeds in response to aflatoxin production. Therefore, 12 samples were used for transcriptome sequencing using Illumina HiSeq2000 system, comprising R and S genotypes with and without inoculation of *A. flavus* and sampled at 1d, 3d and 7d. We performed transcriptomic analysis of the 12 samples i.e., R_CK1, R_CK2, R_CK3, R_T1, R_T2, R_T3, S_CK1, S_CK2, S_CK3, S_T1, S_T2 and S_T3 (where CK is the non-inoculated control, and T indicates inoculated) with two biological replicates, to profile the peanut response to aflatoxin production (Table 2, Additional file 1). We obtained approximately 638.53 million raw reads for the R samples (R_CK1, R_CK2, R_CK3, R_T1, R_T2, and R_T3) and 675.53 million raw reads for the S samples (S_CK1, S_CK 2, S_CK 3, S_T1, S_T2, and S_T3). After filtration of low-quality and adapter sequences, 128.72 Gb of clean bases remained in the 24 transcriptome libraries (Table 2, Additional file 1).

**Table 2:** Summary of the sequence data from Illumina sequencing

Library	Raw reads	Clean reads	Clean bases (Gb)	Error (%)	Q20 (%)	Q30 (%)	GC content (%)
R_CK1_1	57130486	54867978	5.49	0.03	97.20	91.78	44.37
R_CK1_2	54713736	52859150	5.29	0.03	97.40	92.08	45.25
R_T1_1	55671406	55671406	5.57	0.03	97.26	91.79	45.19
R_T1_2	52776632	52776632	5.28	0.03	97.32	91.95	44.87
R_CK2_1	70575134	68662776	6.87	0.03	97.59	92.59	44.90
R_CK2_2	61656004	59697968	5.97	0.03	97.59	92.61	44.86
R_T2_1	62462134	62462134	6.25	0.03	97.66	92.77	44.40
R_T2_2	53206474	53206474	5.32	0.04	96.52	90.20	44.78
R_CK3_1	61917966	59434146	5.94	0.04	96.31	89.28	45.25
R_CK3_2	66649516	63956168	6.40	0.04	96.21	88.97	45.44
R_T3_1	23224306	23224306	2.32	0.05	94.80	87.41	46.27
R_T3_2	18549458	18549458	1.85	0.05	94.71	86.89	46.11
R-Total	638533252	625368596	62.53				
S_CK1_1	58045728	56103334	5.61	0.03	97.24	91.72	45.03
S_CK1_2	63689306	61798978	6.18	0.03	97.32	91.96	44.61
S_T1_1	57998512	57998512	5.80	0.03	97.27	91.85	44.24
S_T1_2	59669162	59669162	5.97	0.03	97.29	91.89	44.35
S_CK2_1	74137916	72280900	7.23	0.03	97.62	92.66	44.80
S_CK2_2	56939790	54259514	5.43	0.04	96.22	89.11	45.46
S_T2_1	49120232	49120232	4.91	0.04	96.27	89.28	44.59
S_T2_2	47405260	47405260	4.74	0.04	96.24	89.28	44.20
S_CK3_1	54341696	51982378	5.20	0.04	96.35	89.38	46.04
S_CK3_2	62599834	59709106	5.97	0.04	96.40	89.89	45.88
S_T3_1	48638388	48638388	4.86	0.04	96.21	89.17	44.96
S_T3_2	42948026	42948026	4.29	0.03	97.23	91.38	44.78
S-Total	675533850	661913790	66.19				

R_CK1, R_CK2, and R_CK3: Zhonghua 6 without inoculated *A. flavus* cultured for 1 day, 3 days, and 7 days, respectively

R_T1, R_T2, and R_T3: Zhonghua 6 with inoculated *A. flavus* cultured for 1 day, 3 days, and 7 days, respectively

S_CK1, S_CK2, and S_CK3: Zhonghua 12 without inoculated *A. flavus* cultured for 1 day, 3 days, and 7 days, respectively

S_T1, S_T2, and S_T3: Zhonghua 12 with inoculated *A. flavus* cultured for 1 day, 3 days, and 7 days, respectively

Q20: The percentage of bases with a Phred value >20

Q30: The percentage of bases with a Phred value >30

**Fig. 1:**
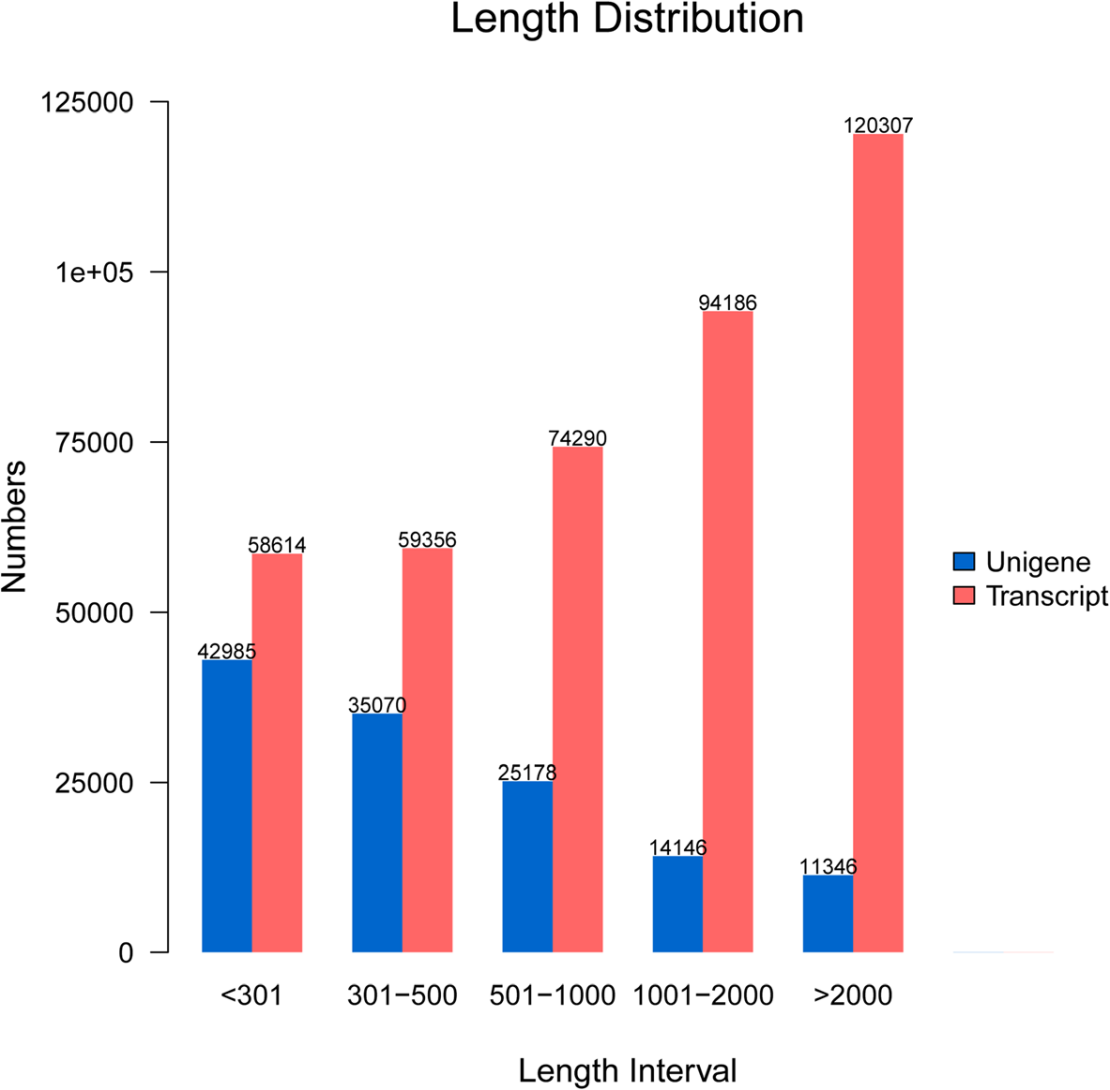
Length distribution of unigenes (*blue*) and transcripts (*red*)

All the high quality reads were then used for *de novo* assembly of transcriptome data using the Trinity software. Using overlapping information in the high-quality reads, 406, 753 transcripts were generated, with an average length of 1, 577 bp and an N50 of 2, 629 bp (Table 3, Fig. 1, and Additional file 2-A). Under the clustering criteria of a minimum of 50 bp overlap and 90 % identity, 128, 725 unigenes were obtained as a comprehensive reference data set of *A. hypogaea* (Table 3); further analysis was based on this final unigene data set. The length of unigenes ranged from 201 to 18, 631 bp, with an average length of 765 bp; unigenes with lengths greater than 500 bp accounted for 39.36 % of all unigenes (Table 3, Fig. 1, and Additional file 2-B).

**Table 3:** Summary of the *de novo* assembly results using Trinity

Category	Number				Total number	Mean length (bp)	N50 value	N90 value	Total nucleotides
	200–500 bp	500–1000 bp	1000–2000 bp	≥2000 bp					
Transcripts	117, 970	74, 290	94, 186	120, 307	406, 753	1, 577	2, 629	755	641, 557, 533
Unigenes	78, 055	25, 178	14, 146	11, 346	128, 725	765	1, 355	293	98, 499, 770

### Gene annotation and functional classification of resistant and susceptible peanut transcriptome

For validation and annotation of the assembled unigenes, all assembled unigenes were first screened against the NCBI non-redundant protein sequences (Nr), NCBI non-redundant nucleotide sequences (Nt), and Swiss-Prot database using the NCBI blast 2.2.28+ program. Among the 128, 725 unigenes, 52, 691 (40.93 %) had significant similarity to 39, 488 unique proteins by Nr analysis. Of all the unigenes, 32, 396 (25.16 %) with significant identities to Swiss-Prot proteins were matched with 17, 871 unique proteins accessions. In addition, 41, 555 (32.28 %) unigenes had matches in the Nt database (Table 4). In total, 62, 352 unigenes (48.43 %) were annotated successfully in at least one of the Nr, Nt, Swiss-Prot, KEGG Ortholog database (KO), Protein family (Pfam), Gene Ontology (GO), and eukaryotic Ortholog Groups (KOG) databases; 7, 061 unigenes (5.48 %) were annotated in all seven databases. However, 66, 373 (51.56 %) unigenes had no matches in those databases. These un-matched unigenes may be novel genes or belong to untranslated regions, and might play specific roles in stress response to aflatoxin production by *A. flavus* in peanut seeds.

**Table 4:** Statistics of the functional annotation of assembled unigenes

Public database	Number of unigenes	Percentage (%)
Nr	52, 691	40.93
Nt	41, 555	32.28
Swiss-Prot	32, 396	25.16
GO	40, 889	31.76
KOG	17, 798	13.82
Pfam	35, 318	27.43
KO	13, 196	10.25
All Databases	7, 061	5.48
Annotated in at least one Database	62, 352	48.43
Total Unigenes	128, 725	100

**Fig. 2:**
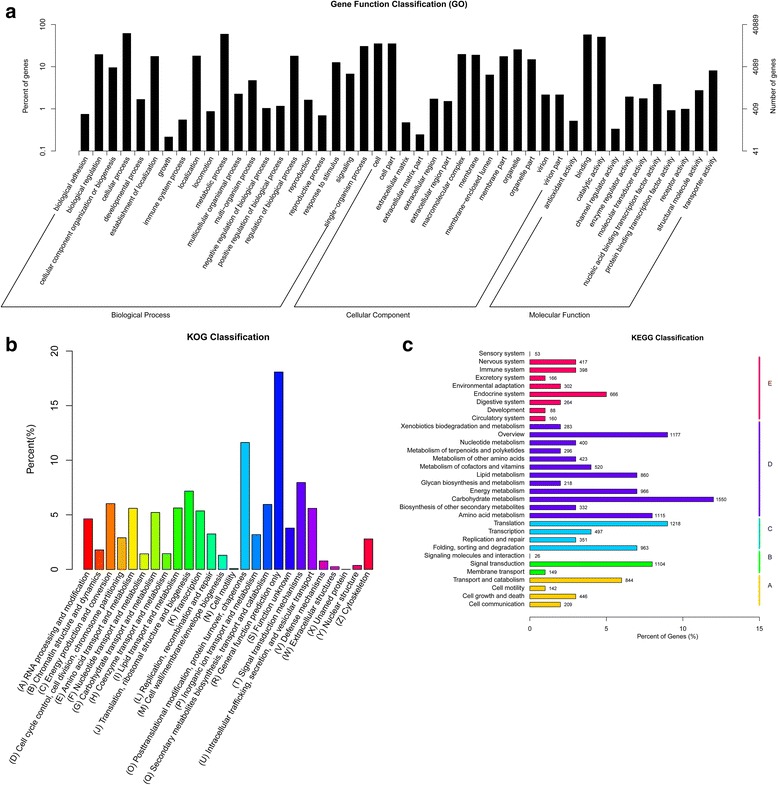
Functional classification of the assembled unigenes. **a** Functional classification of the assembled unigenes based on GO categorization. The results are summarized in the three main GO categories: biological process, cellular components and molecular functions. The x-axis indicates the subcategories, and the y-axis indicates the numbers related to the total number of GO terms present. **b** A histogram of clusters of KOG classification. The unigenes were aligned to the KOG database to predict and classify possible functions. 17, 798 unigenes were annotated and assigned to 26 KOG categories. **c** Pathway assignment based on KEGG database. 13, 196 unigenes were assigned into 32 sub-categories of KEGG pathways under five main categories. A: cellular processes; B: environmental information processing; C: genetic information processing; D: metabolism; E: organismal systems

To identify the functional categories of the annotated unigenes, GO, KOG, and KEGG were used to classify the unigenes annotated by known proteins. In total, 40, 889 unigenes with Blast2GO matches to known proteins were assigned to a broad range of GO terms (Table 4, Fig. 2a, and Additional file 3). The majority of the unigenes were assigned to “Molecular function” (27, 630; 67.57 %), followed by “Biological process” (27, 092; 66.26 %) and “Cellular component” (17, 434; 42.64 %). A total of 17, 798 unigenes were annotated using the KOG database (Table 4), and these unigenes were assigned to 26 KOG categories (Fig. 2b, and Additional file 3). Among the 26 KOG categories, the cluster related to “General function prediction only” (3, 218; 18.08 %) was the largest group, followed by “Posttranslational modification, protein turnover, chaperones” (2, 068; 11.62 %) and “Signal transduction mechanisms” (1, 415; 7.95 %). Additionally, all the unigenes were analyzed with the KEGG pathway database, 13, 196 (10.25 %) with significant matches in the database and were assigned to five main categories, which included 32 sub-categories and 273 KEGG pathways (Table 4, Fig. 2c, and Additional file 3). Among the 32 sub-categories, “Carbohydrate metabolism” was the sub-category with the greatest number of unigenes (1, 550; 11.75 %), followed by “Translation” (1, 218; 9.23 %) and “Amino acid metabolism” (1, 115; 8.45 %). These annotations and classifications provided a valuable resource for investigating specific processes, functions and pathways of the identified unigenes.

### Identification and analysis of differentially expressed genes

Fragments Per Kilobase of transcript sequence per Millions base pairs sequenced (FPKM) was used to quantify the transcript levels of the reads, which facilitated the comparison of mRNA levels both within and between samples [23]. The assembled set of 128, 725 unigenes was used as the reference onto which clean reads from each library were mapped to generate a putative expression profile for the transcripts (Additional file 4). All the 128, 725 unigenes were normalized and calculated by the FPKM method using uniquely mapped reads (Additional files 5 and 6). Unigenes with FPKM value >0.3 were considered to be transcriptionally expressed [24]. Among the unigenes, 93.16 % (119, 917) were expressed in at least one of samples and 19, 230 unigenes were expressed in all 24 libraries (Additional file 5). The expressed unigenes data were highly reproducible between two biological replicates in both R and S genotypes, although a certain number of specifically expressed unigenes were obtained from each biological replicate (Additional files 5 and 7). To validate the RNA-Seq digital expression data, 20 expressed unigenes were randomly selected, primers were designed (Additional file 8) and quantitative real-time reversed transcription PCR (qRT-PCR) was performed. The results showed a high correlation (R^2^ = 0.714; Additional file 9) between the RNA-seq and qRT-PCR data, which confirmed the authenticity of these expressed unigenes and the transcriptome analysis.

Further, DESeq was used to identify the differentially expressed genes (DEGs) across the samples where only those unigenes with the corrected *p* (*q*) value < 0.05 were considered differentially expressed [25]. The differential comparisons between the control and the inoculated samples identified DEGs that responded to aflatoxin production in both genotypes; the comparison between the inoculated samples identified DEG s between the R and S genotypes in response to aflatoxin production (Additional file 10). An important proportion of DEGs (30, 143) were identified in the comparisons among the three time points in both genotypes (Additional files 11 and 12). We observed that the up-regulated and down-regulated DEGs showed similar change trends through the three time points (Additional files 10 and 11). The number of up-regulated DEGs was markedly higher than the down-regulated in comparisons of the control and inoculated samples of both genotypes. There were more up-regulated DEGs in the R genotype than in the S at each time point, while there were fewer down-regulated DEGs in the R genotype than in the S at each time point. To obtain a global view of the gene expression patterns, we performed hierarchical clustering of all the DEGs based on the log_10_ FPKMs for the 12 samples (Additional files 13 and 14). The results showed that the DEGs data were highly reproducible between two biological replicates in both R and S genotypes (Additional file 14). Similar expression patterns were found in the earlier inoculated samples (R_T1 and S_T1); and distinct sample-specific expression patterns were observed in each genotype at the latter two time points (Additional file 13).

### Functional classification of differentially expressed genes

To analyze the functions of the DEGs, a GO analysis was performed using GOseq method in Blast2GO [26]. GO terms with corrected *p* (*q*) value <0.05 were considered significantly enriched among the DEGs. GO enrichment analysis of the up-regulation DEGs in the inoculated sample were compared with the control at paired time points of R and S genotypes, respectively. Many significantly enriched terms in the biological process, molecular function, and cellular component categories were identified (Additional file 15). Metabolic progress (GO:0008152), catalytic activity (GO:0003824), and oxidation-reduction progress (GO:0055114) were dominant terms in comparisons of the *A. flavus*-inoculated (treatment) to non-inoculated (control) (R_T1*vs*. R_CK1, R_T2 *vs*. R_CK2, R_T3 *vs*. R_CK3, S_T2 *vs*. S_CK2, and S_T3 *vs*. S_CK3). Many other common/unique terms affected by aflatoxin production were enriched in the treatments versus the controls of the R and S genotypes, whereas no GO terms were enriched in the comparison of S_T1 *vs*. S_CK1. Notably, the terms antioxidant activity (GO:0016209), phenylpropanoid biosynthetic process (GO:0009699), peroxidase activity (GO:0004601), linoleate 13S-lipoxygenase activity (GO:0016165), oxylipin metabolic progress (GO:0031407), coumarin biosynthetic process (GO:0009805), stilbene biosynthetic process (GO:0009811), cinnamic acid biosynthetic process (GO:0009800) and flavonoid biosynthetic process (GO:0009813), which have key roles in plant resistance to pathogens [27], were exclusively present in the R genotype at the3^rd^ day after incubation (R_T2 *vs*. R_CK2). This indicated that metabolisms involving a series of lipids and secondary metabolites were quite active in the complex resistance processes of the R genotype in response to aflatoxin production. Concurrently, a GO analysis was conducted for the down-regulated DEGs in the inoculated samples of the R and S genotypes, respectively (Additional file 15). However, the analysis failed to confirm enrichment in any term in the down-regulated DEGs data obtained from R_T1 *vs*. R_CK1, S_T1 *vs*. S_CK1, R_T2 *vs*. R_CK2, and S_T2 *vs*. S_CK2. The results suggested that the metabolisms of peanut were activated by *A. flavus* colonization at an early stage of the peanut-*A. flavus* interaction process. When comparing T3 with CK3, protein binding (GO:0005515) and protein folding (GO:0006457) were the dominant GO terms of the down-regulated DEGs in comparisons of both genotypes at the 7^th^ day after incubation. The induction of defense is cost-intensive and contact with pathogens would greatly alter host-plant metabolism [28, 29]. Great changes were observed in gene expression of both R and S genotypes during the peanut -*A. flavus* interaction. Nonetheless, the GO analysis showed more active response in the R genotype than in the S.

To further investigate the biological functions and interactions of genes, pathway-based analysis was conducted using KEGG [30]. All DEGs obtained from the comparisons of the treatment versus the control in R and S genotype at three paired time points were analyzed using KOBAS 2.0 to identify their associated KEGG metabolic pathways [31]. Eighteen pathways were significantly up-regulated in comparisons of the treatment versus the control in both genotypes, while 37 pathways were significantly repressed by aflatoxin production (*q* value < 0.05) (Additional file 16). However, KEGG metabolic pathway analysis failed to confirm enrichment in any up-regulated pathway obtained in R_T3 *vs*. R_CK3, and S_T3 *vs*. S_CK3, which indicated that many metabolic pathways in post-harvest peanut seeds might be repressed by a mass of *A. flavus* mycelia and/or aflatoxin. Not unexpectedly, several up-regulated pathways, such as “phenylpropanoid biosynthesis”, “flavonoid biosynthesis”, and “stilbenoid, diarylheptanoid and gingerol biosynthesis”, were uniquely enriched in the R genotype at the 3^rd^ day after inoculation. This analysis is consistent with the previous observation that the fungus attack can influence a broad range of pathways and a large proportion of the genes in the transcriptional networks are affected [20, 32–35].

### Expression analysis of defense-related genes in peanut response to aflatoxin production by *A. flavus*

Analyzing the expression profiles of R and S genotypes in response to aflatoxin production by *A. flavus*, especially the 30, 143 unigenes that were significantly differentially transcribed (Additional file 11), we detected 842 potential defense-related genes involved in peanut response to aflatoxin production (Additional file 17). These defense-related genes encoded nucleotide binding site-leucine-rich repeat proteins (NBS-LRR), leucine-rich repeat receptor-like kinase (LRR-RLK), mitogen-activated protein kinase (MAPK), transcription factors (TFs), pathogenesis-related (PR) proteins, and crucial factors of other defense related pathways (Additional file 17).

Ninety DEGs with NBS-LRR domains were identified in this study. The *NBS-LRR* genes were all down-regulated at the first time point (1^st^ day after incubation) in R genotype, whereas there were five up-regulated *NBS-LRR* genes in S genotype at the first time point; at the second time point (3^rd^ day after incubation), all *NBS-LRR* genes were up-regulated in both genotypes; about a half of *NBS-LRR* genes in R (54.84 %) and S (55.56 %) were up-regulated at the third time point (7^th^ day after incubation). Overall, the expression levels of *NBS-LRR* genes in R genotype were higher or slightly higher than in S. Furthermore, the expression patterns of 19 DEGs encoding chitinases and 84 DEGs involving in the lectins metabolic pathway were similar to the differentially expressed *NBS-LRR*s. Though the differentially expressed *LRR-RLK*s (143) showed up- or down-regulation in each comparison, there were up-regulated *LRR-RLK*s than down-regulated ones in both genotypes. Interestingly, 28 DEGs involved in MAPK cascades including 6 ones were identified at the first two time points and another 22 ones were obtained at the third time point. Two up-regulated DEGs (comp90525_c1 and comp77989_c0) encoding extracellular signal-regulated kinase 1/2 (K04371) and one up-regulated DEG (comp90797_c0) encoding MAPK kinase 1 (K04368) were identified in both genotypes at the third time point, whose expression level was higher in R genotype than in S. Additionally, 71 *ARF*s (ADP-ribosylation factors), 19 *LOX*s (lipoxygenases) and 6 *PGIP*s (polygalacturonase inhibitor proteins) differentially expressed in the T *vs.* CK, and most of them were up-regulated after *A. flavus* inoculation in both genotypes. Compared with S genotype, 10 *ARF*s, 13 *LOX*s and 6 *PGIP*s were induced to a higher level in the R.

Fifty-eight DEGs encoding WRKY transcription factors (TFs) were identified, and all of them were up-regulated in both genotype. The number of expressed *WRKY*s gradually increased with incubated time in both genotypes, 2 and 21 *WRKY*s were respectively identified at the first and the third time point. The transcript levels of those *WRKY*s were higher in R genotype than in S. As for bZIP TFs, 65 differentially expressed *bZIP*s were identified; however, no *bZIP* was found at the second time point in both genotypes. Two *bZIP*s were identified at the first time point, but there was no significant difference in the expression levels between R and S genotypes. Twenty-three *bZIP*s were found at the third time point, and the transcript levels in R genotype were much higher than in S. Additionally, 67 DEGs encoding ethylene-responsive TFs (ERFs) were identified. All *ERF*s were down-regulated in R or S genotype at the first time point; this down-regulation was more severe in S genotype than in R. Six *ERF*s were up-regulated at the second time point, and 3 of them were identified in both genotypes, which expression levels in S genotype was higher than in R. At the third time point, 43 up- and 22 down-regulated *ERF*s were identified, and 29 of all ones were found in both genotypes.

A total of 86 DEGs were annotated as PR proteins in R and S genotypes, including PR-1, PR-2, PR-5, PR-10, PR-sth2, PR-Bet VI family and other resistance proteins. Thirteen DEGs encoding PR protein Bet VI family and 4 DEGs encoding PR protein STH2 showed greater expression changes in R genotype compared with the S. One DEG (comp91631_c1) annotated as PR-10, was identified in R genotype at the latter two time points, while the DEG was identified in S genotype only at the third time point. Additionally, one DEG was annotated as PR-2 only in R genotype, and the *PR-1* and *PR-5* genes were only identified in S. Most *PR* genes were induced to a higher level in R genotype compared with the S.

We identified 45 DEGs involved in phytohormonal metabolism and signaling pathways that were up- or down-regulated in response to aflatoxin production, including salicylic acid (SA), ethylene (ET), and abscisic acid (ABA). Six DEGs were involved in the SA signaling pathway in both genotypes, and 2 of them (comp80400_c0 and comp91788_c0) encode pathogen-inducible salicylic acid glucosyltransferase (K13691). It is interesting to note that one DEG (comp78095_c0), encoding salicylic acid methyltransferase-like protein (NM_001250193.1), was identified only in R genotype. Moreover, 6 DEGs involving in the ET signaling pathway were identified. Although all ET-related DEGs were down-regulated, most of them were repressed more severely in R genotype than in S. Similarly, 30 DEGs involving in the ABA signal pathway were identified. There were 2, 3 and 3 DEGs respectively encode the key enzymes aldehyde oxidase (AO), 9-cis-epoxycarotenoid dioxygenase (NECD) and Zeaxanthin epoxidase (ZEP) of ABA biosynthesis. *AO*, *NECD* and *ZEP* were up-regulated at the first time point, and down regulated at the third time point in R genotype. While in S genotype, the expression of *AO*, *NECD* and *ZEP* were up-regulated to various extents after *A. flavus* inoculation. Additionally, anther 17 ABA-related DEGs encoding ABA 8′-hydroxylase (7), ABA-insensitive protein (3), ABA receptor (6) and ABA response element binding protein (1) were subjected to up- or down-regulation to various extents in R and S peanut seeds after *A. flavus* colonization.

There were 38 DEGs involving in the biosynthesis of plant phenylpropanoid- derived compounds. Our analysis showed that the expression of DEGs encoding phenylalanine ammonia-lyase (PAL), cinnamate 4-hydroxylase (C4H) and 4-coumarateCoA ligase (4CL) were up-regulated in both genotypes after *A. flavus* inoculation. PAL, C4H and 4CL catalyze the first three steps of phenylpropanoid- derived compounds biosynthesis, the general phenylpropanoid pathway (GPP) [36]. The products of GPP then serve as precursors for diverse phenylpropanoid-derived compounds. Five DEGs encoding PAL were identified in both genotypes that were induced to higher levels in R genotype than in S. Three *PAL*s (comp75395_c0, comp81599_c0, and comp83560_c0) were activated by aflatoxin production at all three time points in R genotype, and their changes in expression levels at the latter two time points were significantly higher than at the first time point. While in comparisons of S genotype, no one and 6 differentially expressed *PAL*s were identified at the first two time points and at the third time point, respectively. Differentially expressed *C4H*s (4) and *4CL*s (9) were identified and showed the same expression patterns as *PAL*s. We also investigated chalcone synthase (CHS), the entry point of the flavonoid pathway, and its close relative stilbene synthase (STS), the key enzyme of stilbenes biosynthesis. In total, 19 DEGs encoding CHS and 3 differentially expressed *STS*s were up-regulated in both genotypes. The expression patterns of *CHS*s and *STS*s were similar to the DEGs encoding the key enzymes of GPP. The results showed that DEGs involving in phenylpropanoid pathway were induced earlier and at a higher level in R genotype compared with the S.

## Discussion

Peanut is an important economic and nutritional crop, and is one of the most susceptible crops to colonization by *A. flavus* and subsequent aflatoxin contamination. A better understanding of molecular mechanism for resistance to aflatoxin contamination will aid designing strategies to develop new peanut cultivars with improved resistance. Transcriptomic analysis is a crucial research approach, as it not only helps in large-scale identification of mRNAs, but also provides insights into the molecular basis of genes involved in plant physiological and pathological processes. In this study, RNA-seq was used to interrogate transcriptome of *A. hypogaea* to explore the molecular mechanism of resistant and susceptible genotypes response to aflatoxin production by *A. flavus*. A large number of *A. hypogaea* transcriptomic unigenes (128, 725) were obtained and about half of the unigenes (62, 352; 48.43 %) were annotated successfully in at least in Nr, Nt, Swiss-Prot, GO, KOG, and KEGG databases. As far as we know, this is the first report to identify large numbers of genes involved in different metabolic pathways in post-harvest peanut seeds in response to aflatoxin production using RNA-seq technology. What’s more, the total clean reads and unigenes, N50 value, and average length of the unigenes reported here were far greater than those previous transcriptomic profiling reports on the developing peanut seed [20, 37, 38]. A large percentage unigenes (51.57 %) could not be annotated in the present study because of technical limitations (such as sequencing depth or read length) [39] and the absence of genomic information on *A. hypogaea* [15], which are common to all studies that perform *de novo* transcriptomic analysis. Transcriptome sequences are valuable resource, especially for species without a completely sequenced genome, such as the cultivated peanut. Our results enriched the genomic information on *A. hypogaea* in public databases, and laid a foundation for the evaluation and understanding of post-harvest peanut seed in response to aflatoxin production.

Previous studies on plant resistance to *A. flavus* infection/aflatoxin production mainly focused on actively developing seeds during the pre-harvest time course [1, 17, 20, 35, 40], and only few reports examined post-harvest peanut seeds [41, 42]. Thus, our study on post-harvest peanut seeds response to aflatoxin production could contribute to a comprehensive understanding of the mechanism of resistance to aflatoxin contamination. In the comparisons analyzed, including among the three time points of both genotypes, we identified 30, 143 DEGs. There were markedly more up-regulated DEGs than down-regulated ones in the comparisons between the treatments versus the controls in both genotypes. In addition, the number of up-regulated DEGs in R genotype was higher than in S at each time point. The results suggested that aflatoxin production activated/repressed the expressions of many genes in R and S genotypes. Genes involved in defensive reactions to aflatoxin production were activated to higher levels in R genotype compared with in S.

Peanut has evolved sophisticated defense mechanisms to combat pathogen invasion, such as blocking pathogen invasion and activating a range of defense responses [22, 43]. *A. flavus* is a facultative parasite that behaves as both a biotroph and a necrotroph [44]. The molecular mechanisms of plant defense against facultative parasites are relatively sophisticated [43]. The mechanism of peanut’s resistance to aflatoxin production was quite complex, with many defense-related DEGs showing transcriptional differences between R and S genotypes in our study. Successful colonization of plant tissues by microbial pathogens requires overcoming the cell wall. To this end, pathogens produce a wide array of plant cell wall degrading enzymes [45]. Polygalacturonases (PGs) cleave the α-(1–4) linkages between the D-galacturonic acid residues of homogalacturonan, causing cell separation in the host tissue. To counteract the activity of PGs, plants deploy cell wall PGIPs that specifically inhibit the pectin-depolymerizing activity of PGs [46]. In addition to PGs inhibition, the interaction between PGs and PGIPs promotes the formation of oligogalacturonides, which are elicitors of a variety of defense responses [47]. Our analysis showed that all 6 differentially expressed *PGIP*s were induced to a much higher level in R genotype than in S, indicating that the PGIPs probably play a more significant role in the defense response to aflatoxin production in R genotype. LRR-RLKs, a large family of signaling proteins comprising extracellular repeats that are linked by a transmembrane domain to either an intracellular adapter domain or a kinase domain [48], take part in a variety of different pathological processes [49]. About 140 differentially expressed *LRR-RLK*s were identified in peanut seeds responding to aflatoxin production by *A. flavus*, and many more *LRR-RLK*s were up regulated in the defensive reactions. FLS2 is a typical pattern recognition receptor (PRR), which can activate the MAPK cascade [50]. Plant MAPK cascades are involved in signaling multiple defense responses, including the biosynthesis and signaling of plant stress and defense hormones, reactive oxygen species (ROS) generation, stomatal closure, defense gene activation, phytoalexin biosynthesis, cell wall strengthening, and the hypersensitive response (HR) cell death [50, 51]. Twenty-eight DEGs involved in MAPK cascades were induced at a higher level in the R genotype after *A. flavus* colonization compared with in S.

Several TFs and other key regulators of plant immunity are induced by the activation of the MAPK cascades [15, 52]. The TFs of the WRKY, bZIP, and ERF families, which have been proven to be involved in plant defense responses [53], were analyzed in this study. The transcription of *WRKY*s is strongly and rapidly up-regulated in response to pathogen invasion and wounding in numerous plant species [54]. Almost all DEGs (58) encoding WRKY proteins were up-regulated, and the activated expression levels of those *WRKY*s were higher in R than in S. Similar to WRKY, the bZIP proteins form a super family of TFs that mediate plant stress responses [55]. Moreover, a bZIP transcription factor, AtfB, is a key player in the coordinated expression of antioxidant genes and genes involved in aflatoxin biosynthesis [56]. We 65 differentially expressed *bZIP*s, but none of them encoded AtfB; however, the transcript levels of 23 *bZIP*s were much higher in R genotype than in S. The ERF family is found only in the plant kingdom, and includes several genes involved in regulation of disease resistance pathways [57]. ERFs probably participate in the regulation of aflatoxin production resistant pathways in peanut seeds similarly to WRKY and bZIP, because most *ERF*s were significantly up-regulated after *A. flavus* inoculation. Additionally, most *ARF*s were up-regulated after *A. flavus* inoculation and 10 of them were up-regulated to higher levels in R compared with in S. The ARFs are family of monomeric GTP-binding proteins belonging to the small GTPases of the Ras super-family, which regulate a wide variety of physiological and pathological processes in various plants [58]. Like all small G-proteins, ARFs functions as molecular switches that alternate between a GTP and membrane-bound “on” state and a GDP-bound, mostly cytosolic “off” state [59]. ARFs might also switch the expression of some target genes involved in peanut’s response to aflatoxin production.

The presence of ABA, SA, and ET phytohormonal pathways in peanut seeds could be concurrent with their response to aflatoxin production by mediating and channeling many stress-responsive genes that help plants to survive stress [60]. ABA is considered as a negative regulator of disease resistance [61, 62]. Consistent with previous reports, the DEGs involved in ABA production and signaling pathway were expressed at high levels in S genotype than in R. Almost all biotic and abiotic stress conditions elicit ET synthesis in plants [63], moreover, ET could inhibit aflatoxin biosynthesis in *A. flavus* on A&M medium [64]. However, all DEGs involved in ET production and signal pathway were down-regulated in response to aflatoxin production, and most of them were repressed to a higher level in R than in S. Depending on the pathogen, disease symptoms seem to be either reduced or enhanced by ET, or not affected, in different plants [63]. We deduced that ET may suppress peanut’s ability to resist aflatoxin production; however, this deduction needs further confirmation. SA plays a crucial role in plants and has a suppressive effect on some fungi [65]. Recent research showed that SA inhibits the mycelial growth and mycotoxin production of *A. flavus* in vitro and in vivo [66]. Six DEGs involved in the SA signaling pathway in both genotypes, and showed similar expression patterns, except for comp78095_c0, encoding salicylic acid methyltransferase-like protein (NM_001250193.1), which was identified only in R genotype. This DEG might be associated with the resistance of R genotype. Phytohormones are involved in mediating fungus-plant interactions, and their roles are totally different [61]. Consistent with previous reports, our transcriptomic analysis showed different expression patterns of genes involved in phytohormone production and signaling in response to aflatoxin production.

*NBS-LRR* genes are the most represented group of plant disease resistance genes which are key component in the interactions between plants and pathogens [43, 67]. Two groups of *NBS-LRR* genes, *CC-NBS-LRR* and *TIR-NBS-LRR*, were identified in both genotypes and showed a general up-regulation after *A. flavus* inoculation. We also found that the expression patterns of those DEGs involved in chitinases and lectins biosynthesis were similar to differentially expressed *NBS-LRR*s. Plant chitinases [68, 69] and lectins [70] are considered to be involved in the plant defense against *A. flavus* [40, 71]. Our results indicated that NBS-LRR, chitinases and lectins probably play important roles in inhibiting aflatoxin production; these DEGs had different responsive reactions between in R and S genotypes. POD proteins, as well as other regulators of peanut immunity [72], contributed to the response to aflatoxin production. Differentially expressed *POD*s showed a significantly higher expression in R compared with in S. The expression of POD, an oxidative radical scavenging enzyme, indicated a better management of oxidative radicals in R genotype during aflatoxin production process. Oxylipins play important roles in the aflatoxin biosynthesis [73]; and 13S-HPODE inhibits aflatoxin production by *A. flavus* [1]. We identified 19 DEGs encoding LOXs and most of them were up-regulated, moreover, “linoleate 13S-lipoxygenase activity”, and “oxylipin biosynthetic process” were enriched in the R genotype. LOXs could affect aflatoxin production [73], and 13-LOXs and their oxidative products could be involved in the defense response to aflatoxin production in the post-harvest peanut seeds.

PR proteins have been defined as proteins encoded by the host plant but induced by various types of pathogens, such as fungi, bacteria, viruses, and also by the application of chemicals that mimic the effect of phytopathogen infection or induce similar stresses [61, 74, 75]. The expressions of PR-1, PR-4 and PR-10 were induced to a higher level to trigger the rapid activation of defense-responsive mechanisms in many fungus-challenged plant species, such as wheat, rice, maize, *Arabidopsis* [61, 76, 77]. Eighty-six DEGs were annotated as PR proteins in R and S genotypes, including PR-1, PR-2, PR-5, PR-10 and other resistance proteins. PR-1 family is induced by SA and pathogens, and is commonly used as a marker for systematic acquired resistance [78]. The PR-2 family consists of β-1, 3-glucanases and catalyzes the hydrolysis of β-1,3-glucans, which act in fungal defense by hydrolyzing fungal cell walls and by generating elicitors [75]. The PR-5 family includes permatins, zeamatins and thaumatin-like proteins, which cause osmotic breakage of transmembrane pores on fungal plasma membranes [75]. PR-10 proteins are small and structurally conserved, but have diverse role in stress signaling [75]. Furthermore, PR-10 proteins have positive roles in maize resistance to *A. flavus* growth and aflatoxin production [76].

Plant phenylpropanoid-derived compounds are a diverse family of phenylalanine-derived secondary metabolites, which include flavonoids, stilbenes, monolignols, and various phenolic acids [79]. Among their many functions in plants, phenylpropanoid compounds play important roles in resistance to pathogen attack [27], moreover, flavonoids and stilbenoids inhibit *A. flavus* development and aflatoxin production [41, 80–82] The key enzymes, PAL, C4H, 4CL, CHS and STS, of the phenylpropanoid biosynthetic pathway were up-regulated in R and S genotypes after *A. flavus* inoculation. The DEGs involving in phenylpropanoid biosynthesis pathway were induced to higher levels in R genotype earlier than inS. In addition, the terms “phenylpropanoid biosynthesis”, “flavonoid biosynthesis”, and “stilbenoid, diarylheptanoid and gingerol biosynthesis” were enriched only in the R genotype. These data suggested that phenylpropanoid-derived compounds biosynthesis might be closely associated with resistance to aflatoxin production in post-harvest peanut seed. Having identified these candidate genes, further research will be required to determine whether these DEGs are responsible for the difference in resistance to aflatoxin production between R and S peanuts.

Although R and S genotypic peanuts both underwent a large transcriptional modulation representing the various metabolic processes involved in defense against aflatoxin production, many more resistance-related DEGs were significantly up-regulated and enriched in R genotype, which suggested that R genotype possessed comprehensive and prompt responses toward the biotic stress. These transcriptional modulations could eventually result in the synthesis of resistance-related proteins, secondary metabolites and signaling molecules that provide defensive advantages to the peanut. Further investigations are necessary to characterize the biosynthesis of these molecules and their molecular mechanisms in response to *A. flavus* colonization and aflatoxin production in the peanut. The comprehensive analysis of their transcriptional profiles under aflatoxin production stress will strengthen the understanding of the genes and metabolic pathways involved in resistance to aflatoxin production, and will provide direction for the future studies on the molecular mechanisms of resistance to aflatoxin contamination in peanut.

## Conclusions

In the present study, RNA-seq was applied to conduct a global characterization of the resistant and susceptible peanut transcriptomes in response to aflatoxin production by *A. flavus*. In total, 128.72 Gb clean bases were obtained and 128, 725 unigenes were assembled from 24 libraries of post-harvest peanut seeds. A number of DEGs were activated or repressed by aflatoxin production in the R and S genotypes, more DEGs were up-regulated in the R genotype than in the S at every time point. Furthermore, 842 putative candidate genes for aflatoxin production resistance in post-harvest seeds were identified. The study provided the first comprehensive report of the transcriptomes of post-harvest peanut seed in response to aflatoxin production, and enhanced the genomic resource database for peanut. Future functional analysis of the responsive genes will provide a better understanding of the molecular mechanism of defense against aflatoxin contamination in peanut and will facilitate identifying major candidate genes and molecular markers for improving resistance to aflatoxin contamination.

## Methods

### Plant material and treatments

Seeds of Zhonghua 6 and Zhonghua 12 were obtained from the Oil Crops Research Institute of Chinese Academy of Agricultural Sciences (CAAS-OCRI). Previous experiments showed that both Zhonghua 6 and Zhonghua 12 were susceptible to seed invasion by *A. flavus* in post-harvest seeds; however, their resistance to aflatoxin production was highly different, with Zhonghua 6 being resistant and Zhonghua 12 susceptible [41]. The toxigenic *A. flavus* strain (AF2202) isolated from peanut was maintained in 20 % glycerol (−80 °C) at CAAS-OCRI. To prepare the *A. flavus* inoculation, conidia of AF2202 were taken from the stored sample and cultured on fresh potato dextrose agar medium at 29 ± 1 °C for 7 days. Conidia were then collected and suspended in sterile water containing 0.05 % Tween-80. The concentration of conidia in the suspension was determined using a haemocytometer.

Healthy post-harvest mature seeds of the R and S genotypes were selected for the experiments. All seeds were surface-sterilized by immersion in 70 % ethanol for 1.0 min, and rinsed with sterile distilled water three times for 5.0 min each. In the artificial inoculation treatments, 0.5 ml spores suspension (4.0 × 10^6^ CFU/ml) was directly added to 10.0 g of peanut seeds in a sterile Petri plate. In the control, 0.5 ml 0.05 % Tween-80 solution was added to the peanut seeds. Then, the inoculated samples and the control were placed in an incubator and cultured at 29 ± 1 °C in darkness. Depending on the specific purpose of the experiment, the seeds were taken out to test aflatoxin content (five replications) or to extract RNA (two replications) after incubation for 1 to 10 days.

The incubated peanut seeds autoclaved at 121 °C for 30 min, then dried at 110 °C for 60 min. After cooling, the seeds of each experimental unit (10 g) were finely ground into powders, then were extracted using 50.0 ml methanol–water (55:45; v/v) in a flask. After shaking extraction (200 rpm, 30 min) and filtration with quantitative filter paper, the filtrate (10.0 ml) was collected in a 125 ml flask, then diluted with 90 ml methanol–water (55:45), blended and filtered with organic membrane (0.45 μm). The purified extract (1.0 ml) was collected in the glass tube and 10.0 μl was prepared for high performance liquid chromatography (HPLC) analysis [5, 82]. HPLC analysis was performed with an Agilent 1200 HPLC system (USA) equipped with a fluorescence detector (G1321A) at wavelengths of 360 nm and 440 nm for excitation and emission, respectively. Chromatographic separation was performed on a C_18_ column (150 mm × 4.6 mm, 5 μl particle size), with a methanol-water (45:55) mobile phase, at a flow rate of 0.7 ml/min.

### RNA isolation and cDNA library construction

Seeds of the R and S genotypes inoculated with *A. flavus* (treatment) and without inoculation (control) cultured for 1, 3 and 7 days were sampled for RNA isolation and cDNA library construction. Two replicates were prepared for each sample, resulting in 24 libraries that were used for transcriptome sequencing using the Illumina HiSeq2000 system at Novogene Bioinformatics Technology Co. Ltd., (Beijing, China).

Total RNA of post-harvest peanut seeds was isolated using the RNeasy® Plant Mini Kit (QIAGEN), according to the manufacturer’s protocol. All RNA samples were treated with RNase-free DNase I. The concentration and integrity of the pooled total RNA was checked using a NanoDrop® 2000 spectrophotometer, a Qubit® Fluorometer 2.0, and an Agilent 2100 bioanalyzer, to confirm that all samples had an RNA integrity number greater than 6.5. RNA quality detection, cDNA library construction, and Illumina deep sequencing were performed following the previous method [15, 18–20].

### Data filtering and de novo sequence assembly

Raw data (raw reads) in the fastq format were first processed using in-house perl scripts. The raw data were then filtered by data-processing steps to generate original clean data via a process that included the removal of adapters, reads containing poly-N and low quality reads. For the samples infected with *A. flavus*, the original clean data contained a certain amount of *A. flavus*’s transcriptomic data. All paired-end clean reads were aligned to the reference genome of *A. flavus* using Tophat (v2.0.7) with “mismatch 2” as the parameter [83], then the transcriptomic data of *A. flavus* were filtered out and to obtaind clean data of *A. hypogaea*. The calculation of Q20, Q30, GC content and the sequence duplication level, and all downstream analyses were used the clean data with high quality. After the clean data were generated, the assembled *A. hypogaea* reference genome was processed using Trinity software with min_kmer_cov set to 1 and other parameters set to default values [84].

### Functional annotation of unigenes

For functional annotation, all assembled unigenes were annotated based on the following seven databases: Nr, Nt, KOG, Swiss-Prot, Pfam, GO, and KO. The unigenes were annotated in Nr, Nt and Swiss-Prot databases using NCBI blast 2.2.28+, with an E-value of 1.0 × 10^−5^, and annotated in KOG database using NCBI blast 2.2.28+ with an E-value of 1.0 × 10^−3^. KAAS (rl40224) was applied to annotate the unigenes in the KO database with a cutoff E-value of 1.0 × 10^−10^ [85]. The unigenes annotated in the Pfam database using HMMScan (HMMER 3) with an E-value of 0.01 [86]. In addition, the unigenes were assigned GO annotations using Blast2go (b2g4pipe_v2.5) with an E-value of 1.0 × 10^−6^ [87].

### Expression analysis and enrichment analysis

Gene expression levels were estimated using the RNA-Seq with Expectation-Maximization (RSEM) (rsem-1.2.0) method for each library [24]. The index of the assembled reference genome was built using Bowtie (mismatch 2), and clean reads of each library were aligned to the assembled reference genome using RSEM. RSEM then counted the read numbers mapped to each gene. Then, the FPKM of each gene was calculated based on the length of the gene and read count mapped to it [83].

Differential expression analysis of two samples was performed using the DESeq R package (1.12.0). DESeq provide statistics to determine differential expression in gene expression data using a model based on the negative binomial distribution. The resulting *p* values were adjusted using the Benjamini and Hochberg’s approach to control the false discovery rate. In this study, unigenes with an adjusted *p* (*q*) value <0.05 found by DESeq were considered as differentially expressed [25].

GO enrichment analysis of the DEGs was conducted using GOseq R packages based on Wallenius non-central hyper-geometric distribution [26], which can adjust for gene length bias in DEGs. GO terms with corrected *p* (*q*) value <0.05 were considered significantly enriched among the DEGs. KEGG pathway database records the network of molecular interactions in the cells and variants specific to particular organisms, with molecular information mainly from large-scale molecular datasets generated by genome sequencing and other high-throughput experimental technologies [30]. KOBAS (v2.0.12) software was used to enrich the DEGs in the KEGG pathways [88]. A corrected *p* (*q*) value <0.05 was the threshold for significantly enriched KEGG pathways in this study.

### qRT-PCR analysis

To validate the repeatability and reproducibility of gene expression data obtained by RNA-seq in *A. hypogaea*, we randomly selected 20 unigenes for validation by qRT-PCR, as described previously [15, 18–20]. Independent RNA of the control peanut seeds without *A. flavus* inoculation of R and S genotypes, which were incubated for 1, 3, and 7 days at 29 ± 1 °C, was prepared for qRT-PCR analysis. RNA extraction and quality control were performed as described above. Gene-specific primer pairs (Additional file 8) were designed according to the sequences of the 20 genes, using the GenScript Real-time PCR Primer Design program available online (https://www.genscript.com/ssl-bin/app/primer). To ensure accuracy, each primer was run with three replications on the same plate, with a negative control that lacked template cDNA to detect non-specific products. Candidate genes were tested in triplicate wells and in three replicate experiments. The relative expression levels of the genes were calculated using the 2^−ΔΔCt^ method [89, 90], which represents the C_T_ (cycle threshold) difference between the reference *Actin* gene and the target gene product [15].

## Availability of supporting data

The sequencing data generated in this study have been deposited in NCBI’s Short Read Archive database (SRA, http://www.ncbi.nlm.nih.gov/Traces/sra_sub/sub.cgi) and are accessible through SRA series accession number SRP061959 (BioProject ID: PRJNA291797).

## Additional files

Additional file 1:
**Classification of raw reads.** Raw reads including clean reads (purple), adapter sequences (green), reads containing undefined nucleotides (N’s) (yellow), and low quality reads (orange) generated from Illumina RNA-sequencing (RNA-seq). (RAR 1485 kb) Additional file 2:
**The length distribution of unigenes and transcripts.** (A) Transcript length distribution; (B) Unigene length distribution. (RAR 97 kb) Additional file 3:
**Summary of GO, KOG, and KEGG classifications of the assembled unigenes.** (XLSX 32 kb) Additional file 4:
**Summary of the uniquely mapped reads in each library.** (XLSX 10 kb) Additional file 5:
**Distribution of unigenes expression in each library.** NEGs: the number of expressed unigenes in each library. NSEGs: the number of specifically expressed unigenes in each library. (DOCX 17 kb) Additional file 6:
**Statistics analysis of FPKM value of all unigenes.** (XLSX 16479 kb) Additional file 7:
**Pearson correlation analysis between samples.** (RAR 473 kb) Additional file 8:
**Primer information for genes used to validate the FPKM-based expression data using qRT-PCR.** (XLSX 10 kb) Additional file 9:
**Quantitative real-time PCR validations of the FPKM-based expression of unigenes by RNA-seq.** Note: RE indicates relative expression levels of unigenes by qRT-PCR. (RAR 18 kb) Additional file 10:
**Comparison analysis of differentially expressed genes.** (DOCX 16 kb) Additional file 11:
**Differentially expressed unigenes analysis and annotation in each comparison.** (XLSX 16087 kb) Additional file 12:
**Number of DEGs in the control and** ***A. flavus*** **inoculated samples of peanut seeds.** The numbers of DEGs in one sample or comparison are shown in each circle. The numbers of DEGs between the two samples or comparisons are shown in the overlapping regions. (RAR 118 kb) Additional file 13:
**Hierarchical clustering of the differentially expressed unigenes in the peanut seeds.** The blue, white, and red bands indicate low, intermediate, and high gene expression quantity, respectively. (RAR 2787 kb) Additional file 14:
**Hierarchical clustering of the DEGs in two biological replicates of the peanut seeds.** A, B and C showed the results of hierarchical clustering analysis at three culture time points, respectively. The blue, white, and red bands indicate low, intermediate, and high gene expression quantity, respectively. (RAR 110 kb) Additional file 15:
**GO functional enrichment analysis of differentially expressed genes when peanut seeds respond to aflatoxin production by** ***A. flavus.*** (XLSX 78 kb) Additional file 16:
**KEGG pathway enrichment analysis of differentially expressed genes when peanut seeds respond to aflatoxin production by** ***A. flavus.*** (XLSX 12 kb) Additional file 17:
**Expression analysis of peanut defense-related genes in response to aflatoxin production by** ***A. flavus.*** (XLSX 373 kb)
