# Supplementary material for: Comparative transcript profiling of resistant and susceptible peanut post-harvest seeds in response to aflatoxin production by Aspergillus flavus
Source: BMC Plant Biol. 2016 Feb 27;16:54. doi: 10.1186/s12870-016-0738-z (PMC4769821; doi:10.1186/s12870-016-0738-z)
Supplement: Additional file 5: — Distribution of unigenes expression in each library. NEGs: the number of expressed unigenes in each library. NSEGs: the number of specifically expressed unigenes in each library. (DOCX 17 kb) [file 12870_2016_738_MOESM5_ESM.docx]

**Additional file 5 - Distribution of unigenes expression in each library**

| **FPKM value** | **Number of unigenes** | | | | | | NSEGs | NEGs |
| --- | --- | --- | --- | --- | --- | --- | --- | --- |
|  | **0-0.1** | **0.1-0.3** | **0.3-3.57** | **3.57-15** | **15-60** | **>60** |  |  |
| R_CK1_1 | 76335 | 9035 | 26756 | 7652 | 6122 | 2825 | 694 | 43355 (33.68%) |
| R_CK1_2 | 78908 | 9375 | 24643 | 7033 | 5900 | 2866 | 556 | 40442 (31.42%) |
| R_T1_1 | 84068 | 9015 | 19843 | 6539 | 6260 | 3000 | 234 | 35642 (27.69%) |
| R_T1_2 | 83956 | 8398 | 20337 | 6584 | 6370 | 3080 | 278 | 36371 (28.25%) |
| S_CK1_1 | 71063 | 10259 | 30151 | 7905 | 6392 | 2955 | 503 | 42005 (32.63%) |
| S_CK1_2 | 71278 | 10326 | 29540 | 8184 | 6415 | 2982 | 1529 | 52145 (40.51%) |
| S_T1_1 | 79982 | 9100 | 22822 | 6969 | 6695 | 3157 | 3758 | 44152 (34.30%) |
| S_T1_2 | 76843 | 9290 | 25314 | 7340 | 6745 | 3193 | 501 | 41289 (32.08%) |
| R_CK2_1 | 76910 | 9810 | 25796 | 7002 | 6302 | 2905 | 391 | 41146 (31.96%) |
| R_CK2_2 | 67357 | 9223 | 33840 | 8950 | 6461 | 2894 | 577 | 45065 (35.01%) |
| R_T2_1 | 73287 | 11286 | 26871 | 7702 | 6684 | 2895 | 1805 | 51978 (40.38%) |
| R_T2_2 | 75998 | 11438 | 24136 | 7502 | 6793 | 2858 | 1133 | 49140 (38.17%) |
| S_CK2_1 | 79969 | 9547 | 22767 | 7010 | 6485 | 2947 | 883 | 47403 (36.83%) |
| S_CK2_2 | 79085 | 8712 | 24508 | 7254 | 6259 | 2907 | 934 | 47121 (36.61%) |
| S_T2_1 | 75120 | 11991 | 24703 | 7657 | 6532 | 2722 | 359 | 39643 (30.80%) |
| S_T2_2 | 64640 | 11527 | 33571 | 8551 | 7177 | 3259 | 428 | 42592 (33.09%) |
| R_CK3_1 | 78770 | 8809 | 24751 | 7215 | 6145 | 3035 | 488 | 39209 (30.46%) |
| R_CK3_2 | 71969 | 11691 | 28067 | 7762 | 6214 | 3022 | 822 | 40928 (31.79%) |
| R_T3_1 | 72675 | 4072 | 29055 | 13489 | 6902 | 2532 | 493 | 41614 (32.33%) |
| R_T3_2 | 76518 | 3067 | 26631 | 13162 | 6807 | 2540 | 371 | 52558 (40.83%) |
| S_CK3_1 | 75805 | 8879 | 27717 | 7747 | 5765 | 2812 | 732 | 44041 (34.21%) |
| S_CK3_2 | 74721 | 9339 | 28180 | 7841 | 5827 | 2817 | 775 | 44665 (34.70%) |
| S_T3_1 | 71194 | 11995 | 28152 | 7667 | 6773 | 2944 | 503 | 45536 (35.37%) |
| S_T3_2 | 70536 | 12557 | 28582 | 7562 | 6670 | 2818 | 673 | 45632 (35.45%) |
| Total number of expressed unigenes | | | | | | | | 119917 (93.16%) |
| Number of expressed unigenes in all libraries | | | | | | | | 19230 |

NEGs: the number of expressed unigenes in each library.

NSEGS: the number of specifically expressed unigenes in each library.
