# Supplementary material for: Comparative transcript profiling of resistant and susceptible peanut post-harvest seeds in response to aflatoxin production by Aspergillus flavus
Source: BMC Plant Biol. 2016 Feb 27;16:54. doi: 10.1186/s12870-016-0738-z (PMC4769821; doi:10.1186/s12870-016-0738-z)
Supplement: Additional file 10: — Comparison analysis of differentially expressed genes. (DOCX 16 kb) [file 12870_2016_738_MOESM10_ESM.docx]

**Additional file 10 - Comparison analysis of** **differentially expressed genes**

| Comparison | Number of DEGs | | |
| --- | --- | --- | --- |
|  | Up-regulated | Down-regulated | Total |
| R_T1 vs R_CK1 | 990 | 601 | 1, 591 |
| R_T2 vs R_CK2 | 779 | 102 | 881 |
| R_T3 vs R_CK3 | 13, 838 | 4, 003 | 17, 841 |
|  |  |  |  |
| S_T1 vs S_CK1 | 945 | 788 | 1, 733 |
| S_T2 vs S_CK2 | 2, 423 | 0 | 2, 423 |
| S_T3 vs S_CK3 | 10, 382 | 7, 218 | 17, 600 |
|  |  |  |  |
|  |  |  |  |
| R_T1 vs S_T1 | 785 | 479 | 1, 264 |
| R_T2 vs S_T2 | 106 | 217 | 323 |
| R_T3 vs S_T3 | 11, 236 | 5, 834 | 17, 070 |
|  |  |  |  |
| R_T2 vs R_T1 | 819 | 972 | 1, 791 |
| R_T3 vs R_T2 | 10, 014 | 994 | 11, 008 |
| R_T3 vs R_T1 | 12, 244 | 2, 522 | 14, 766 |
|  |  |  |  |
| R_CK2 vs R_CK1 | 13 | 153 | 166 |
| R_CK3 vs R_CK2 | - | 6 | 6 |
| R_CK3 vs R_CK1 | 1, 150 | 2, 455 | 3, 605 |
|  |  |  |  |
|  |  |  |  |
| S_T2 vs S_T1 | 2, 455 | 110 | 2, 565 |
| S_T3 vs S_T2 | 8 | 2 | 10 |
| S_T3 vs S_T1 | 7, 095 | 3, 444 | 10, 539 |
|  |  |  |  |
| S_CK2 vs S_CK1 | 341 | 1, 517 | 1, 858 |
| S_CK3 vs S_CK2 | 858 | 391 | 1, 249 |
| S_CK3 vs S_CK1 | 2, 082 | 2, 922 | 5, 004 |
| Total | 30, 143 | | |
